# Supplementary material for: Genome-wide siRNA screens identify RBBP9 function as a potential target in Fanconi anaemia-deficient head-and-neck squamous cell carcinoma
Source: Commun Biol. 2023 Jan 13;6:37. doi: 10.1038/s42003-022-04389-3 (PMC9839743; doi:10.1038/s42003-022-04389-3)
Supplement: Supplementary file 2 — Supplementary Information file [file 42003_2022_4389_MOESM2_ESM.pdf]

Supplementary Table 1: Characteristics of cell lines

Cell Line	type	FA-compl. group	FA mutation	Mean chromosome number	TP53 status	TP53 Mutation	Origin	Gender	Use in this study
VU-SCC1131	HNSCC	C	Homozygous c.67delG/c.67delG	74,38 ± 4,98	inactivated	R273L	Floor of mouth	F	Primary screen and Validation
VU-SCC1131 +FANCC (Lentiviral correction of FANCC)	HNSCC,	n.a.	Homozygous c.67delG/c.67delG	74,38 ± 4,98	inactivated		Floor of mouth	F	Primary screen and Validation
VU-SCC1365	HNSCC	A	Homozygous c.3788_3790delTCT/ c.3788_3790delTCT	63,66 ± 7,4	inactivated	R282W	mouth mucosa	M	Primary screen and Validation
VU-SCC1604	HNSCC	L	Heterozygous c.483delATCAC/ c.891C>G	66,76 ± 5,7	inactivated	V216Gfs*5	tongue	F	Primary screen and Validation
RPE-1 hTERT wt	Diploid, normal		n.a.	46	wt	wt	ATCC	F	Validation
RPE-1 hTERT FANCA-/-	Diploid, normal	A	Biallelic out-of-frame deletion	46	wt	wt	CRISPR-Cas9 engineered	F	Validation
ARPE-19 hTERT wt	Diploid, normal	n.a	n.a.	46	wt	wt	ATCC, hTERT immortalized in-house	M	Validation
ARPE-19 hTERT FANCC -/-	Diploid, normal	C	Biallelic out-of-frame deletion	46	wt	wt	CRISPR-Cas9 engineered	M	Validation
ARPE-19 hTERT FANCA -/-	Diploid, normal	A	Biallelic out-of-frame deletion	46	wt	wt	CRISPR-Cas9 engineered	M	Validation
VU-1131 patient-matched CAFs, hTERT	Diploid, normal	C	Homozygous c.67delG/c.67delG	46	wt	wt	Tumour Biopsy	F	Validation
VU-1131 patient-matched CAFs, SV40 large T	Diploid	C	Homozygous c.67delG/c.67delG	46	inactivated	n.a	Tumour Biopsy	F	Validation
VU-1365 patient-matched Fs, hTERT	Diploid, normal	A	Homozygous c.3788_3790delTCT/ c.3788_3790delTCT	46	wt	wt	Skin	M	Validation
VU-1604 patient-matched CAFs, hTERT	Diploid, normal	L	Heterozygous c.483delATCAC/ c.891C>G	46	wt	wt	Tumour Biopsy	F	Validation
VU-1604 patient-matched CAFs, SV40 large T	Diploid	L	Heterozygous c.483delATCAC/ c.891C>G	46	inactivated	n.a	Tumour Biopsy	F	Validation
ARPE-19 hTERT RBBP9 -/-	Diploid, normal	n.a	Biallelic out-of-frame deletion	46	wt	wt	CRISPR-Cas9 engineered	M	Validation and functional characterisation

wt – wild-type, CAFs = Cancer-associated fibroblasts, Fs = Skin fibroblasts, M – male, F – female, n.a. – not applicable.

Supplementary Figure 1

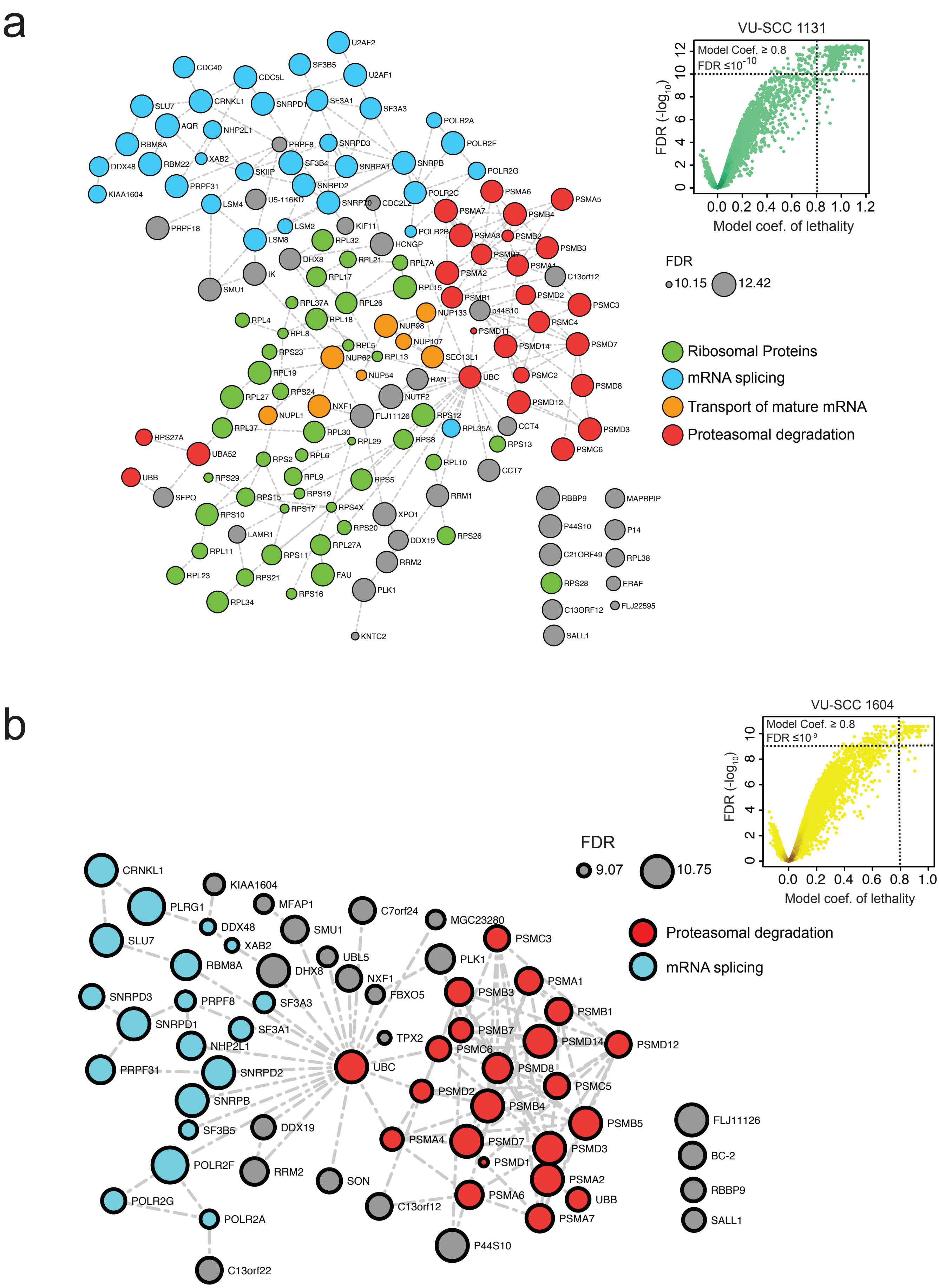

Supplementary Figure 1. Genetic essentiality and interaction networks in in Fanconi Anaemia head and neck squamous cell carcinoma.

Gene essentiality, interaction network and pathway prediction analysis of lethal vulnerabilities in the **a)** VU-SCC 1131 (FA-C) and **b)** VU-SCC 1604 (FA-L) HNSCC cell lines. Lethality scores (model coefficient of lethality) were calculated with respect to the non-targeting siRNA control. Model coefficient and FDR cut-offs used are indicated in the volcano plots. Genetic interactions are mined from GeneMania database and pathway predictions are generated using Reactom. Nodes and edges represent gene names and known physical interactions respectively. Nodes sizes are scaled to -log10 FDR values. Node colours depict pathway associations.

Supplementary Figure 2

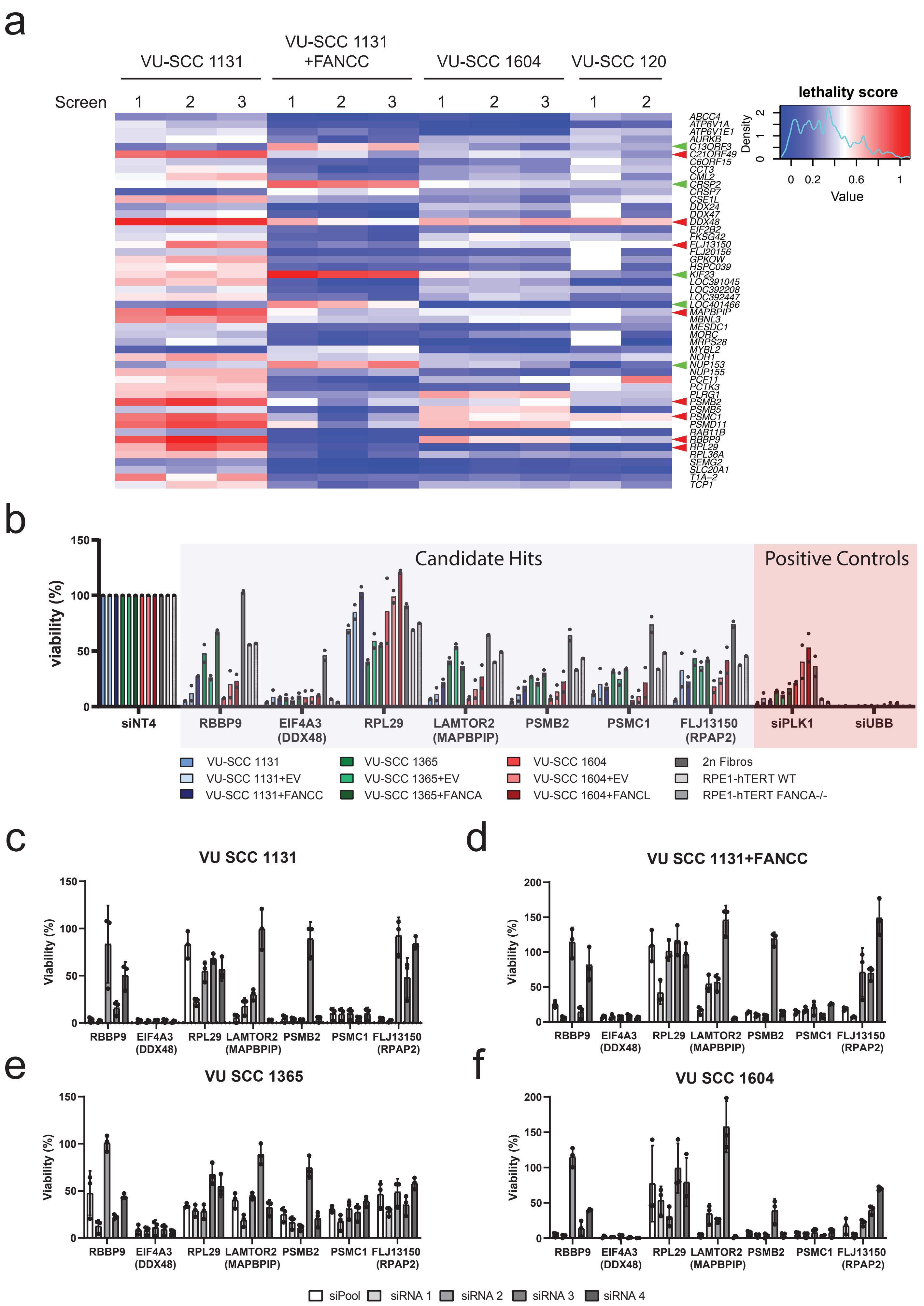

Supplementary Figure 2. Extended validation and hit selection.

a) Heat map depicting top 50 differentially lethal genes. Red arrows indicate candidate genes enriched for FA-HNSCC specific lethality shown in **b**, green arrows exemplify candidate genes whose silencing exerts the opposite outcome, i.e., lethal to FA pathway reconstituted HNSCC cell line. Published data from a sporadic HNSCC cell line, VU-SCC 120, previously screened using the same library and screening platform (n=2) was also included in the analysis so as to enrich for FA-HNSCC specific candidates.

b) Validation of candidate hits discovered in the primary siRNA screen of FA-HNSCC tumour cell lines (Fig.2a, b), in an independent, extended panel of appropriately complemented FA-HNSCC isogenic cell lines - (VU-SCC 1131 + EV and VU-SCC 1131 + FANCC, VU-SCC 1365 + EV and VU-SCC 1365 + FANCA, VU-SCC 1604 + EV and VU-SCC 1604 + FANCL) along with their respective parental FA-HNSCC lines (VU-SCC 1131, VU-SCC 1365, VU-SCC 1604) as well as normal cells - human diploid hTERT fibroblasts, RPE1 hTERT WT and RPE1 hTERT FANCA-/-. All siRNAs were used at a concentration of 10nM in the validation experiments. CellTiter-Blue® was used as readout for viability. Fluorescence values were normalised to untreated controls of the respective cell line. EIF4A3, RPL29 and FLJ13150/RPAP2 were inconsistent with the primary screen and hence dropped. Note the trend of FA-HNSCC specificity of RBBP9, LAMTOR2, PSMB2 and PSMC1, i.e., less lethal in FA-pathway proficient counterparts of the cell line pairs. RBBP9 is the only non-essential gene among these candidates. n=2 independent experiments each in technical duplicates. Controls - NT = non-targeting, UBB = Ubiquitin B, PLK1 = Polo-like kinase 1.

C - f) Deconvolution was performed by scoring the efficacy of four individual siRNAs targeting a gene of interest in the FA-HNSCC cell lines VU-SCC 1131 (FA-C) **c**, VU-SCC 1131+FANCC **d**, VU-SCC 1365 (FA-A) **e**, and VU-SCC 1604 (FA-L) **f**. When at least two of four single siRNAs reproduced the lethal effect of their cognate siRNA pools in the screened cell lines, the candidate gene was considered a genuine hit. Cell viability as measured by CellTiter Blue was normalised to the non-targeting siRNA control. A hit was considered lethal when cell viability was less than 40%. n=3 independent experiments. Error bars = SD.

Supplementary Figure 3

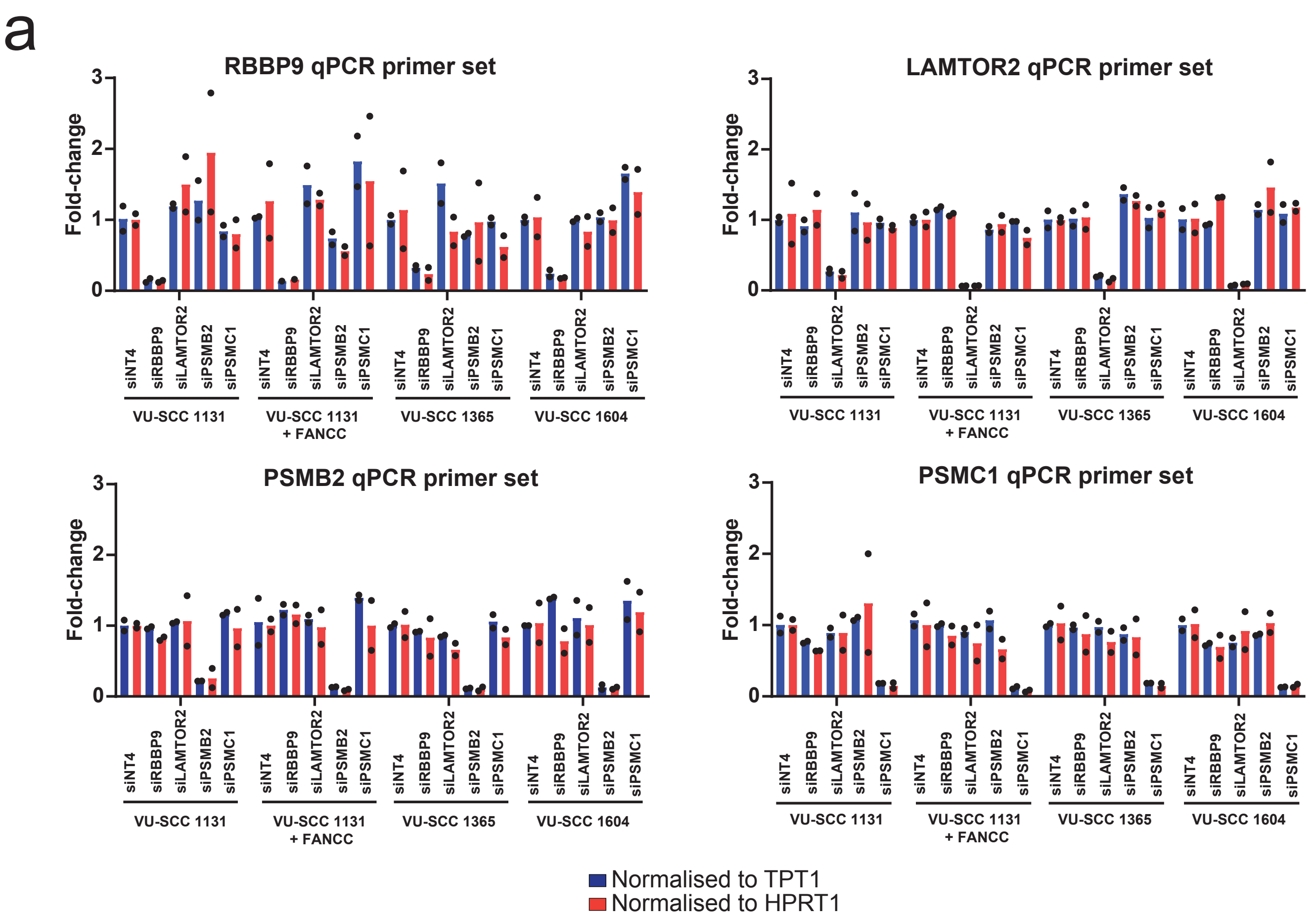

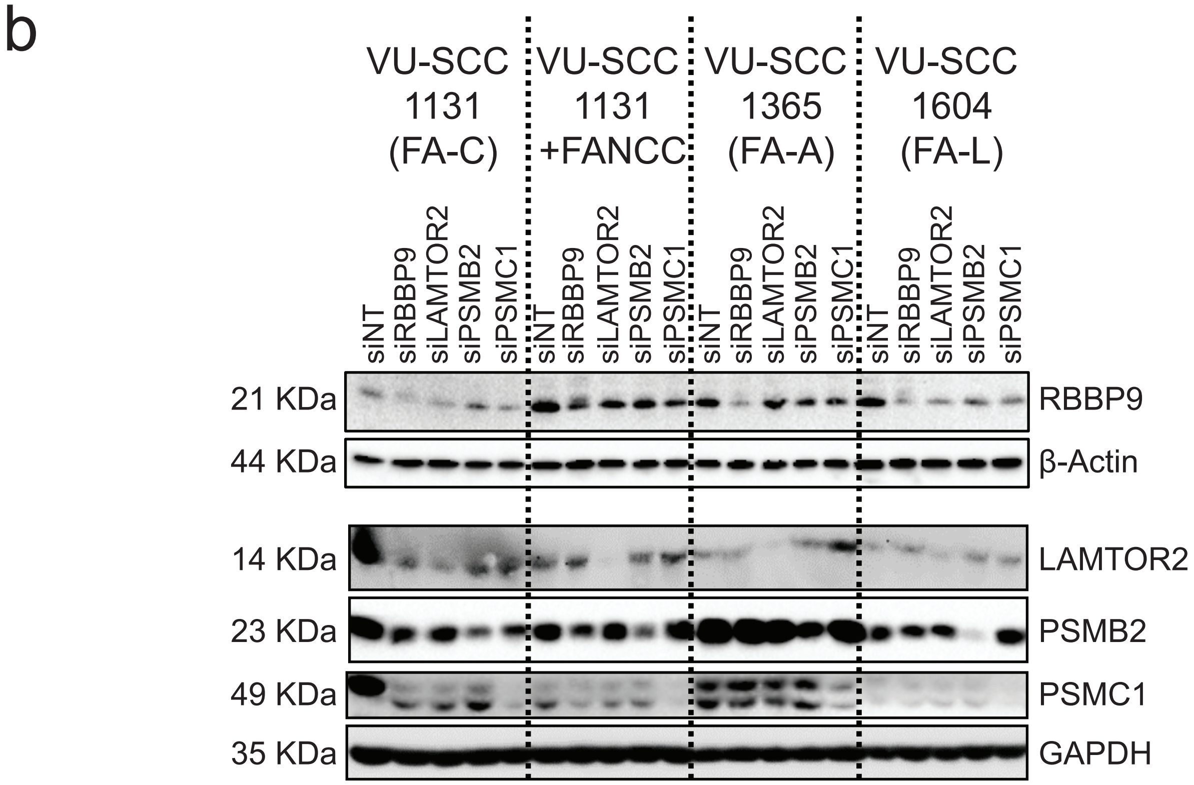

Supplementary Figure 3. SMARTpool siRNA specificity and efficiency.

a) Quantitative RT-PCR of all four validated targets confirming mRNA depletion after 24h of SmartPOOL siRNA (10nM) knockdowns in FA-HNSCC tumour cell lines. These experiments were performed once, in technical duplicates and normalised to expression levels of two different reference/housekeeping genes. See also **b**. Housekeeping controls TPT1 = Tumour Protein, Translationally-Controlled 1, HPRT1 = Hypoxanthine Phosphoribosyltransferase 1.

b) Representative immunoblots of samples treated as in **a** (10nM siRNA, 24h), confirming knockdown efficiency at the protein level. Note the general, expected difference in abundance of target proteins between the patient-derived FA-HNSCC cell lines.

Supplementary Figure 4

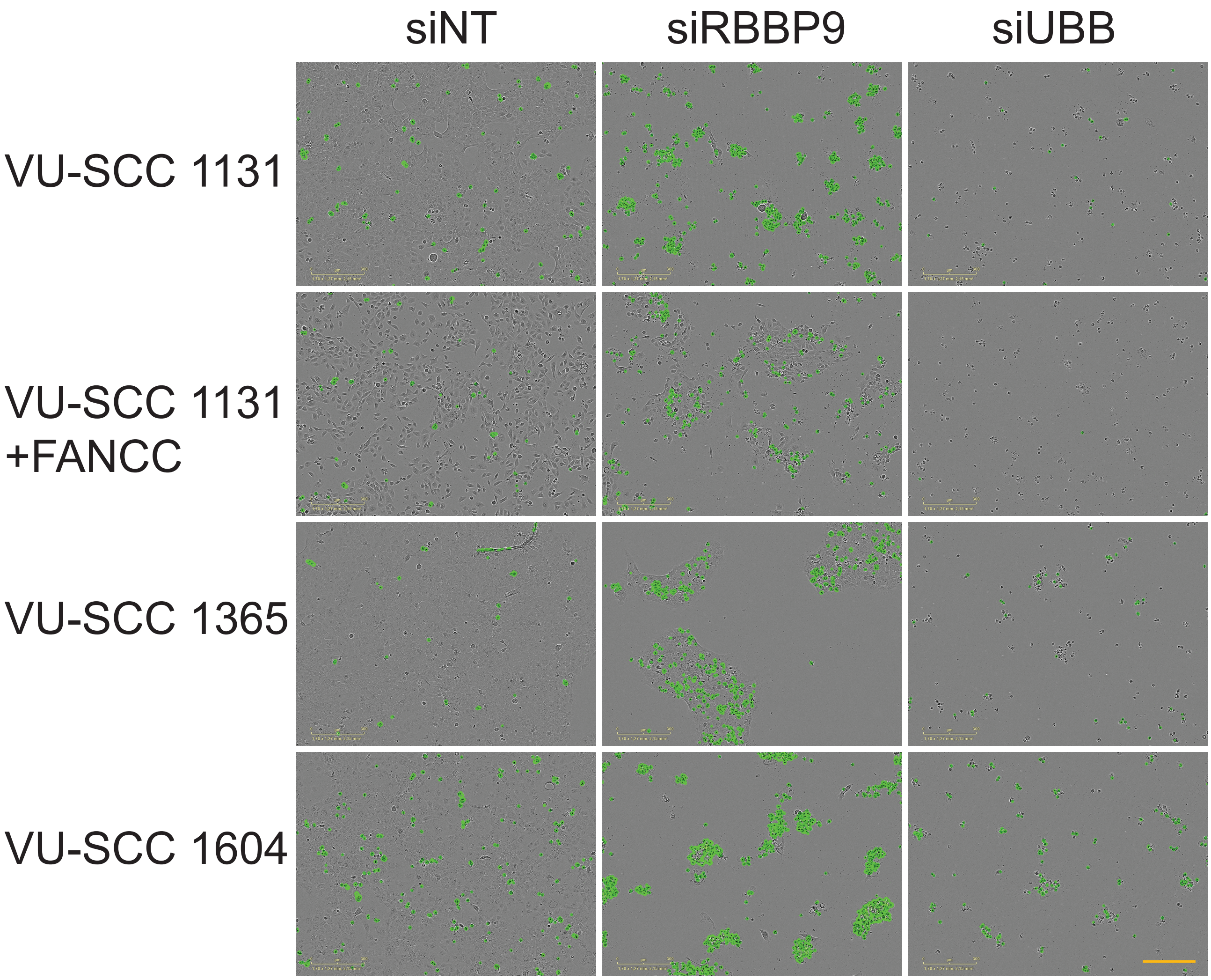

Supplementary Figure 4. RBBP9 silencing induced Caspase-3-mediated apoptosis.

Figure depicts complete (wider) frames of photomicrographs (at 72h) in Fig 3d, of live FA-HNSCC cells IncuCyte®-imaged in 96-well plates for up to 100h after RBBP9 knockdown, fed with the Caspase 3 fluorogenic substrate NucView 488®. Green fluorescent cells represent apoptosis. Scale bars (yellow) = 300µm.

Supplementary Figure 5

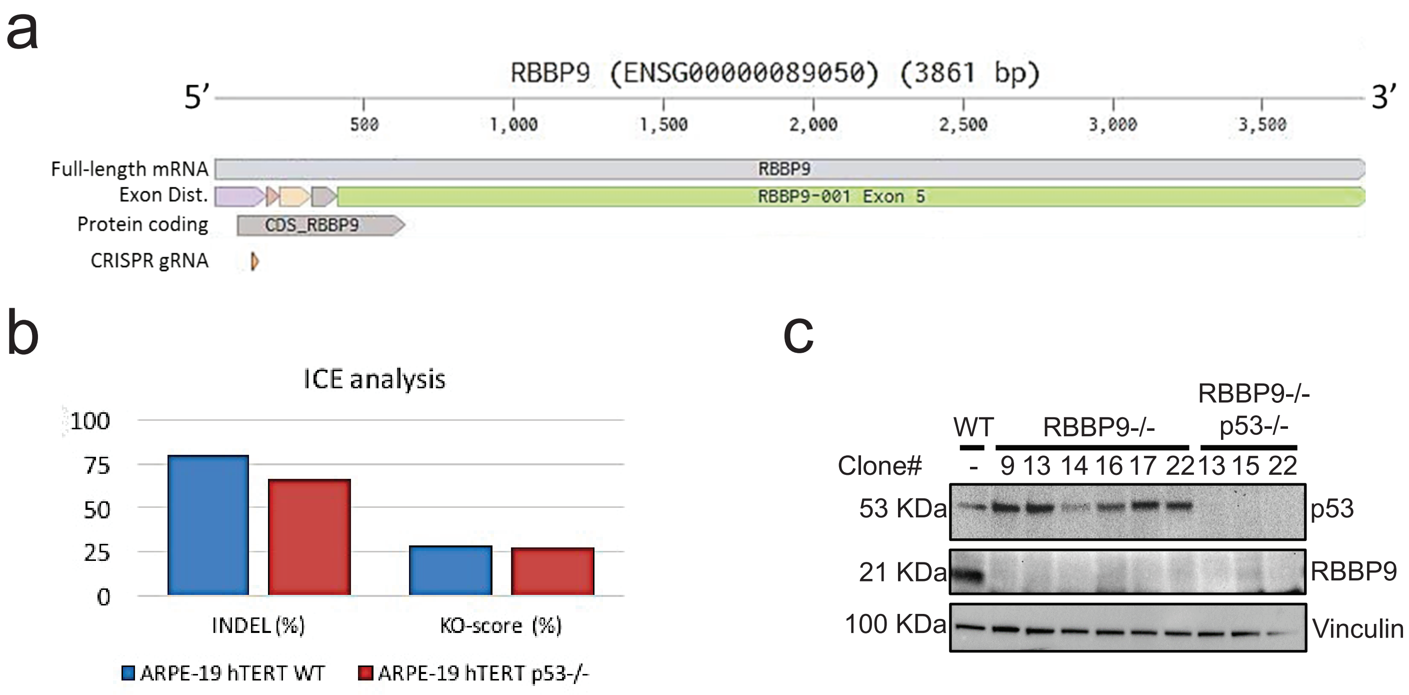

Label	Guide Target	PAM sequence	INDEL (ICE)	Model Fit (R^2)	KO-Score
ARPE-19 hTERT WT	GTGACCACCCACGGCTGGTA	TGG	80	0,97	28
ARPE-19 hTERT p53-/-	GTGACCACCCACGGCTGGTA	TGG	66	0,96	27

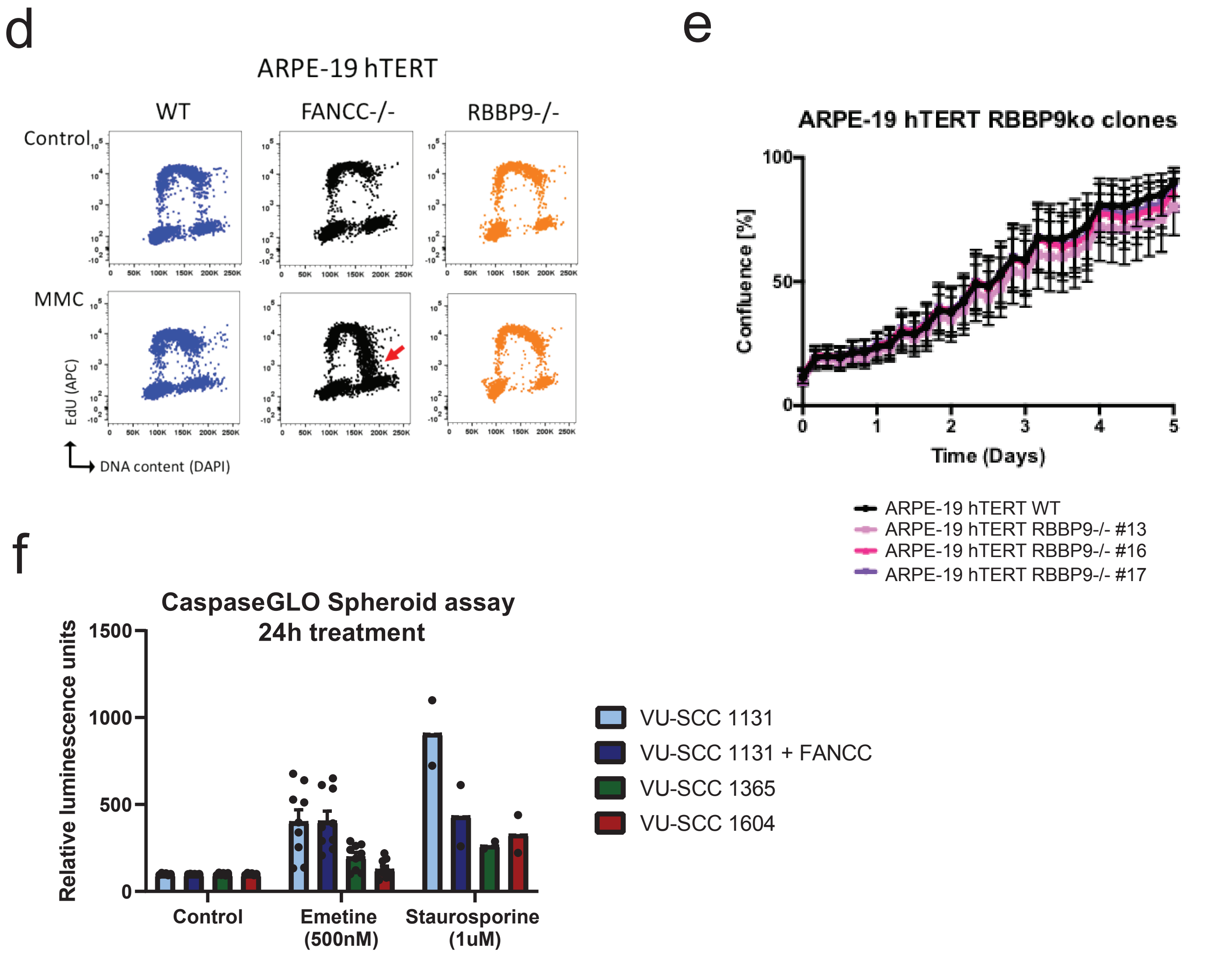

Supplementary Figure 5. Generation and characterization of RBBP9-/- ARPE19-hTERT cells.

a) Schematic representation of the full-length, human RBBP9 transcript, with exon distribution (5 exons), protein-coding transcript segment and positioning of the synthetic guide RNA (CRISPR gRNA) within exon 1. gRNA sequence provided in **b**.

b) RBBP9 Gene targeting efficiency in the CRISPRed cell pools (ARPE-19 hTERT WT and ARPE-19 hTERT p53^{-}/$^{-}$) as determined by shallow-sequencing of PCR amplicon of target region and synthego ICE analysis (https://ice.synthego.com/#/) after 1 week of Cas9:crRNA:tracrRNA ribonucleoprotein nucleofection. See methods for details. % Indels = sum total insertion and deletion mutations at target locus. % Knockout score = predicted loss of gene function due to relative large (> 21 bp) deletions at target locus.

c) Immunoblot of single cell clones derived from WT and p53^{-}/$^{-}$ ARPE-19 hTERT cell lines, showing loss of RBBP9 protein expression in several clones. See also Supplementary Figure 8.

d) Representative EdU-incorporation cell cycle distribution flow cytometry plots of WT, FANCC^{-}/$^{-}$, and RBBP9^{-}/$^{-}$ ARPE-19 hTERT cell lines. Note the typical late-S/G2 arrest of mitomycin C (MMC)-treated FANCC^{-}/$^{-}$ cells, and the lack thereof, in RBBP9^{-}/$^{-}$ cells. suggesting that RBBP9 loss does not sensitise cells to DNA damage.

e) IncuCyte® cell growth curves of 3 independent clones (#13, #16, #17 shown in c) of RBBP9^{-}/$^{-}$ ARPE-19 hTERT cells showing no apparent change in cell growth properties due to loss of RBBP9, suggesting RBBP9 loss or inhibition is well tolerated by normal diploid human cells. n=2 independent experiments in duplicates. Error bars = SD.

f) Caspase-GLO® luminescent apoptosis assay (Caspase-3 and -7 cleavage-based) of FA-HNSCC 3D spheroid models after Emetine-treatment (500nM, 24h). Staurosporine (1uM, 4h) was used to induce apoptosis as a positive control. See also Fig. 4d. n=3 independent experiments. Error bars = SD.

Supplementary Figure 6

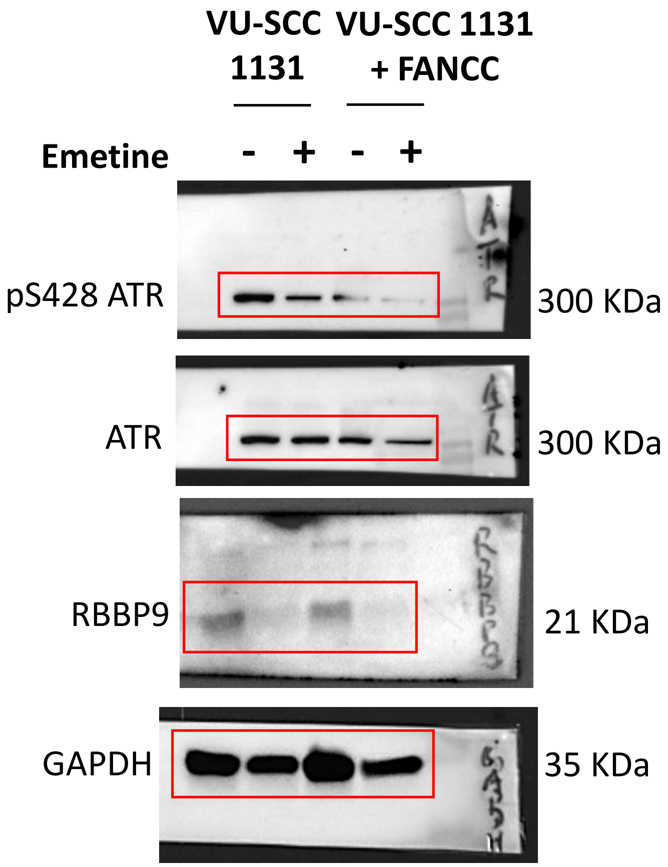

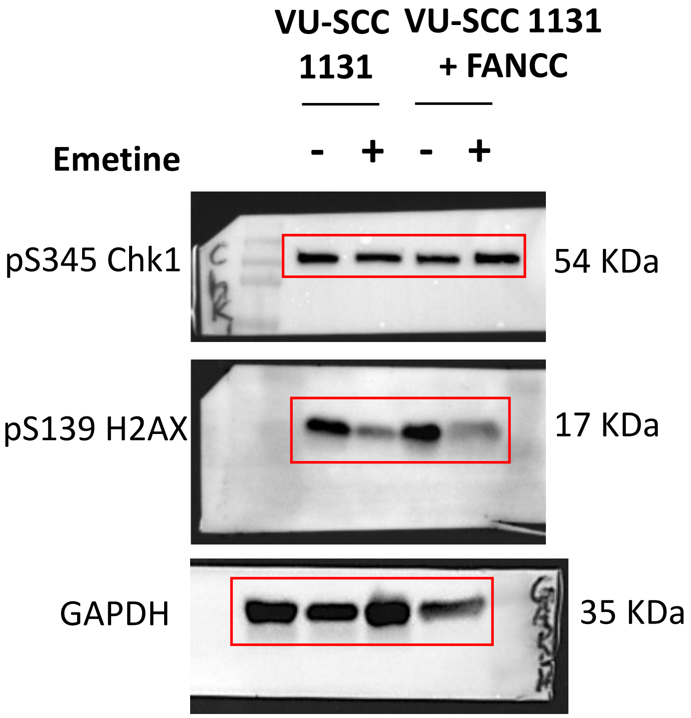

Precision plus™ protein dual color standard, BioRad # 1610374

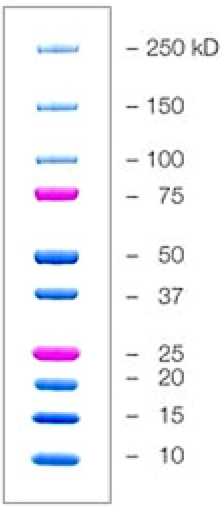

Supplementary Figure 6. Emetine does not cause DNA damage at low dose in the FANCC-deficient VU-SCC 1131 cell line.

Immunoblots pertaining to Figure **4e**, of the isogenic VU- SCC 1131 (FA-deficient) and VU-SCC 1131 + FANCC (FA-proficient) cell-line pair treated with 25nM Emetine for 72h. Red boxes indicate cropped area presented in Figure **4e**. Levels of the DNA damage marker pS139 H2AX, and the activity of replication checkpoint kinases pS428 ATR and pS345 Chk1, collectively suggest that low-dose Emetine treatment induces cell death independent of DNA damage. GAPDH was used as loading control.

Supplementary Figure 7

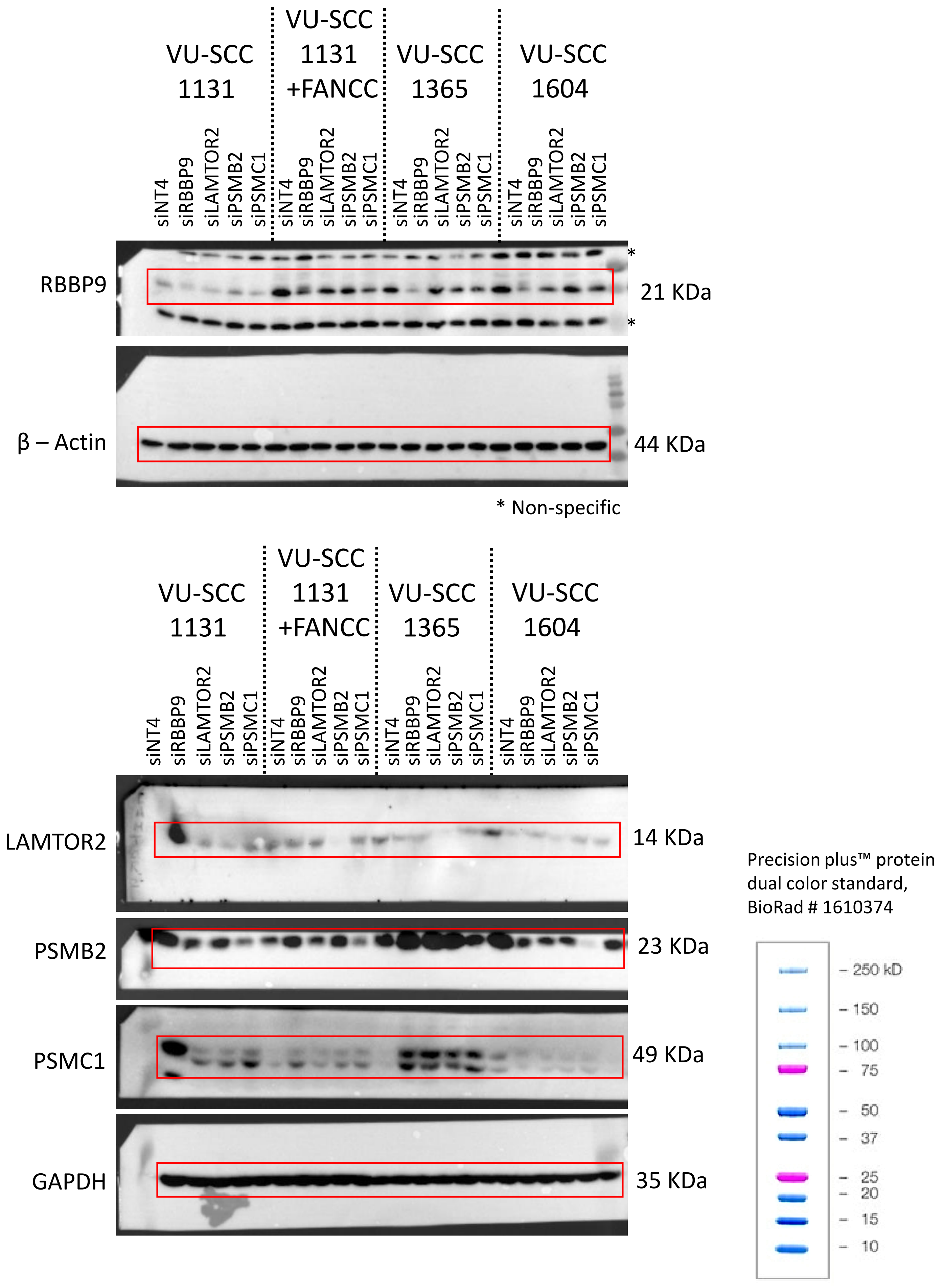

Supplementary Figure 7. Depletion of protein targets after siRNA knockdown of validated hits.

Immunoblots pertaining to Supplementary Figure **3b** after SmartPOOL siRNA (10nM) knockdowns (24h) of all four validated targets in FA-HNSCC tumour cell lines from the original screen confirming knockdown efficiency at the protein level. Note the general, expected difference in abundance of target proteins between the patient-derived FA-HNSCC cell lines. Red boxes indicate cropped area presented in Supplementary Figure **3b.** * indicates non-specific bands. β – Actin and GAPDH were used as loading controls.

Supplementary Figure 8

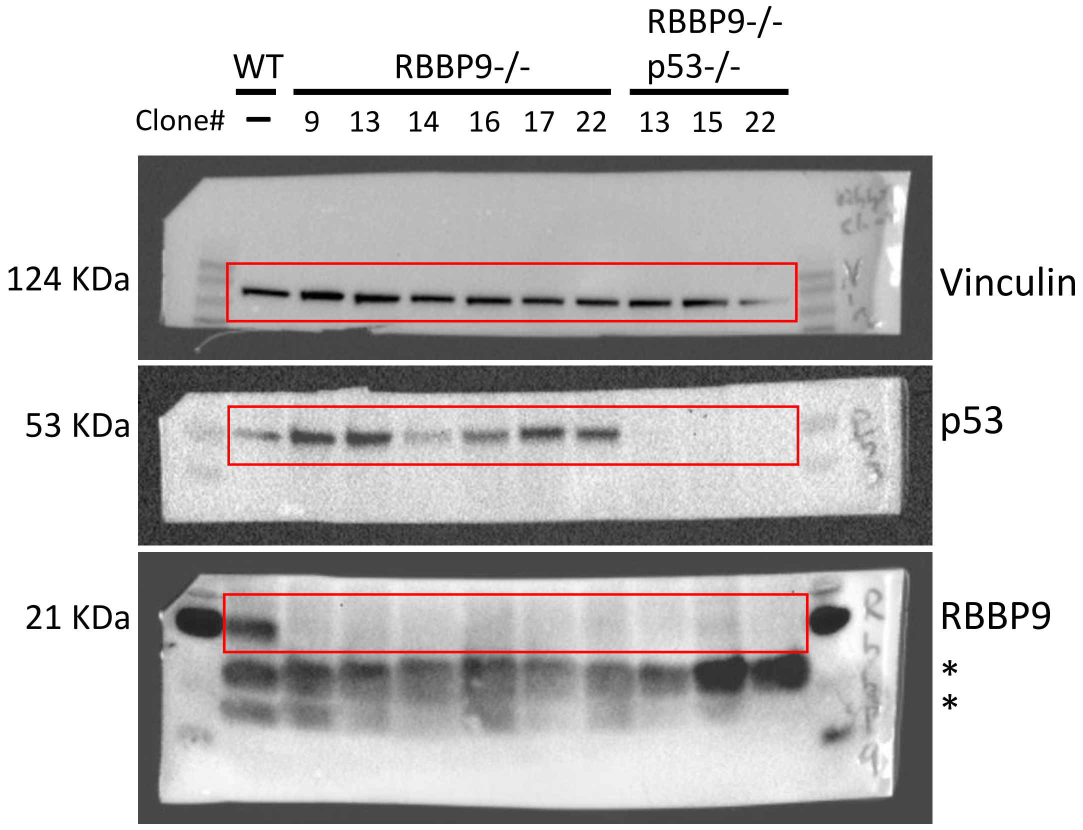

* non-specific bands

Precision plus™ protein dual color standard, BioRad # 1610374

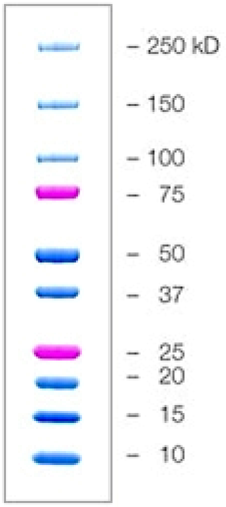

Supplementary Figure 8. Screening RBBP9 CRISPR/Cas9-knockout clones

Immunoblots pertaining to Supplementary Figure **5c**, of single cell clones derived from WT and $p53^{-}/^{-}$ ARPE-19 hTERT cell lines, showing loss of RBBP9 protein expression in several clones. Knockouts were generated using a Cas9-RNP complex approach. See methods section for details. Red boxes indicate cropped area presented in Supplementary Figure **5c.** * indicates non-specific bands. Vinculin was used as loading control.

Supplementary Figure 9

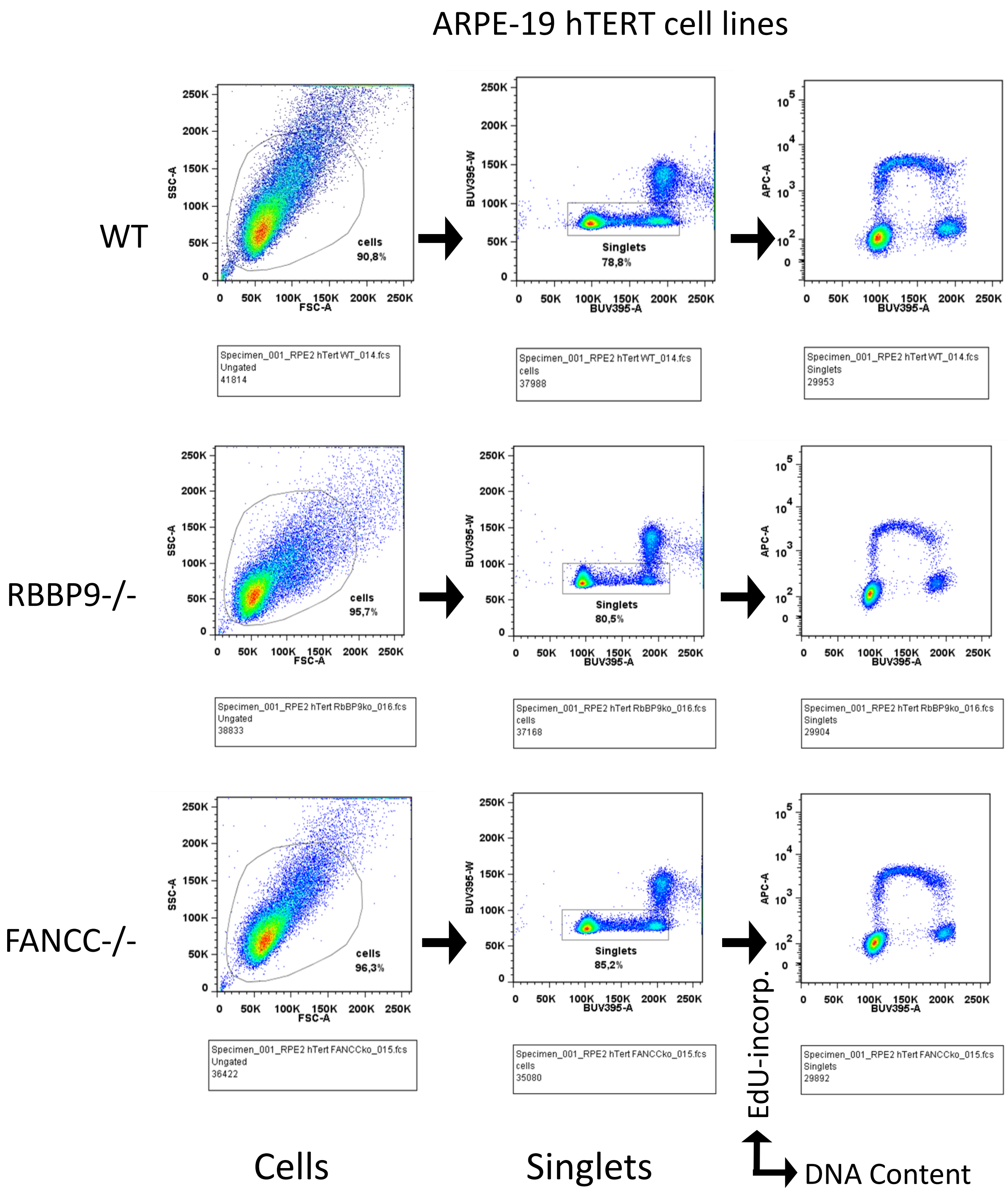

Supplementary Figure 9. Gating strategy for EdU-incorporation cell cycle flow cytometry.

For EdU-incorporation cell cycle analysis pertaining to Supplementary Figure **5d**, single cells were gated from the cell population by plotting DNA content area versus width i.e, DAPI-A (area; X-axis) against DAPI-W (width; Y-axis). Dotplots presented in Supplementary Figure **5d** show at least 20000 single cells with DNA content (DAPI-A, X-axis) vs EdU labelling (APC-A, Y-Axis).
