# Supplementary material for: Genome-wide siRNA screens identify RBBP9 function as a potential target in Fanconi anaemia-deficient head-and-neck squamous cell carcinoma
Source: Commun Biol. 2023 Jan 13;6:37. doi: 10.1038/s42003-022-04389-3 (PMC9839743; doi:10.1038/s42003-022-04389-3)
Supplement: Supplementary file 3 — Description of Additional Supplementary Files [file 42003_2022_4389_MOESM3_ESM.pdf]

Description of Additional Supplementary Files

Supplementary data 1: Normalised screen data excel file.

Supplementary data 2: RBBP9-FLAG MSbased interactome data.

Supplementary data 3: siRNA Screen data in relation to CRISPR screen genetic studies on HNSCC.

Supplementary data 4: Experimental source data file
