# Supplementary material for: Genome-wide siRNA screens identify RBBP9 function as a potential target in Fanconi anaemia-deficient head-and-neck squamous cell carcinoma
Source: Commun Biol. 2023 Jan 13;6:37. doi: 10.1038/s42003-022-04389-3 (PMC9839743; doi:10.1038/s42003-022-04389-3)
Supplement: Supplementary file 8 — Reporting Summary [file 42003_2022_4389_MOESM8_ESM.pdf]

nature research

Corresponding author(s): M. G. Pai

Last updated by author(s): Aug 16, 2022

Reporting Summary

Nature Research wishes to improve the reproducibility of the work that we publish. This form provides structure for consistency and transparency in reporting. For further information on Nature Research policies, see our Editorial Policies and the Editorial Policy Checklist.

Statistics

For all statistical analyses, confirm that the following items are present in the figure legend, table legend, main text, or Methods section.

n/a	Confirmed	
☐	☒	The exact sample size (*n*) for each experimental group/condition, given as a discrete number and unit of measurement
☐	☒	A statement on whether measurements were taken from distinct samples or whether the same sample was measured repeatedly
☐	☒	The statistical test(s) used AND whether they are one- or two-sided<br>*Only common tests should be described solely by name; describe more complex techniques in the Methods section.*
☒	☐	A description of all covariates tested
☐	☒	A description of any assumptions or corrections, such as tests of normality and adjustment for multiple comparisons
☐	☒	A full description of the statistical parameters including central tendency (e.g. means) or other basic estimates (e.g. regression coefficient) AND variation (e.g. standard deviation) or associated estimates of uncertainty (e.g. confidence intervals)
☐	☒	For null hypothesis testing, the test statistic (e.g. F, t, r) with confidence intervals, effect sizes, degrees of freedom and P value noted<br>*Give P values as exact values whenever suitable.*
☒	☐	For Bayesian analysis, information on the choice of priors and Markov chain Monte Carlo settings
☐	☒	For hierarchical and complex designs, identification of the appropriate level for tests and full reporting of outcomes
☒	☐	Estimates of effect sizes (e.g. Cohen's *d*, Pearson's *r*), indicating how they were calculated

Our web collection on statistics for biologists contains articles on many of the points above.

Software and code

Policy information about availability of computer code

Data collection	Not applicable.
Data analysis	Not applicable.

For manuscripts utilizing custom algorithms or software that are central to the research but not yet described in published literature, software must be made available to editors and reviewers. We strongly encourage code deposition in a community repository (e.g. GitHub). See the Nature Research guidelines for submitting code & software for further information.

Data

Policy information about availability of data

All manuscripts must include a data availability statement. This statement should provide the following information, where applicable:

- Accession codes, unique identifiers, or web links for publicly available datasets
- A list of figures that have associated raw data
- A description of any restrictions on data availability

-The mass spectrometry proteomics data have been deposited to the ProteomeXchange Consortium via the PRIDE partner repository (http://www.ebi.ac.uk/pride) with the dataset identifier PXD026545.

-List of figures that have associated raw data:
-Figure 1, 4F.
-Supplementary Figure 1.

-No restrictions on data availability.

Field-specific reporting

Please select the one below that is the best fit for your research. If you are not sure, read the appropriate sections before making your selection.

☒ Life sciences ☐ Behavioural & social sciences ☐ Ecological, evolutionary & environmental sciences

For a reference copy of the document with all sections, see nature.com/documents/nr-reporting-summary-flat.pdf

Life sciences study design

All studies must disclose on these points even when the disclosure is negative.

Sample size	15 cell lines.
Data exclusions	No data excluded.
Replication	Unless otherwise mentioned, all screens, validation and other experiments were performed in at least three independent biological replicates.
Randomization	Not applicable.
Blinding	Not applicable.

Reporting for specific materials, systems and methods

We require information from authors about some types of materials, experimental systems and methods used in many studies. Here, indicate whether each material, system or method listed is relevant to your study. If you are not sure if a list item applies to your research, read the appropriate section before selecting a response.

Materials & experimental systems

n/a	Involved in the study	
☐	☒	Antibodies
☐	☒	Eukaryotic cell lines
☒	☐	Palaeontology and archaeology
☒	☐	Animals and other organisms
☒	☐	Human research participants
☒	☐	Clinical data
☒	☐	Dual use research of concern

Methods

n/a	Involved in the study	
☒	☐	ChIP-seq
☐	☒	Flow cytometry
☒	☐	MRI-based neuroimaging

Antibodies

Antibodies used	RBBP9 (Abcam, Cat# ab-157202, Santa Cruz, Cat# sc-101111), LAMTOR2 (Cell Signaling, Cat# 8145S), PSMB2 (Enzo Life Sciences, Cat# BML-PW9300-0100), PSMC1 (Bethyl labs, Cat# A303-821A), P-ATR (S248) rabbit Ab (Cell Signaling, Cat# 2853S), ATR (N-19), Goat polyclonal IgG (SantaCruz, Cat# sc-1887), P-Chk1 (S345) (133D3) (Cell Signaling, Cat# 2348S), Chk1 (2G1D5) mouse mAb (Cell Signaling, Cat# 2360S), pS139 H2AX JBW-301 (Upstate/Millipore, Cat# 05-636), pS139 H2AX (Novus Biologicals, Cat# NB100-384), Histone H3 (Cell Signaling, Cat# 9715), β–Actin mAb (Santa Cruz, Cat# sc-47778), β Tubulin (D-10) (Santa Cruz, Cat# sc-5274), Vinculin (H-10) (Santa Cruz, Cat# 25336). HRP-conjugated goat anti-rabbit (Dako, Cat# P044801-2), goat anti-mouse (Dako, Cat# P044701-2) or Rabbit anti-goat (Dako, Cat# P044901-2).
Validation	Standard antibodies have been used. For the RBBP9 antibody, the performed siRNA and KO experiments showed the specificity of the antibody.

Eukaryotic cell lines

Policy information about cell lines

Cell line source(s)	Previously published FA-patient-derived cell lines generated in house, ATCC.
Authentication	No authentication performed.
Mycoplasma contamination	Cell lines were routinely outsourced to Microbiome T.a.v, Amsterdam for PCR-based mycoplasma testing and confirmed to be negative.
Commonly misidentified lines (See ICLAC register)	Not applicable.

Flow Cytometry

Plots

Confirm that:

☒ The axis labels state the marker and fluorochrome used (e.g. CD4-FITC).

☒ The axis scales are clearly visible. Include numbers along axes only for bottom left plot of group (a 'group' is an analysis of identical markers).

☒ All plots are contour plots with outliers or pseudocolor plots.

☒ A numerical value for number of cells or percentage (with statistics) is provided.

Methodology

Sample preparation	For PI exclusion cell death assays, 30000 – 50000 cells were seeded per well in 24-well plates, transfected with 10nM siRNAs or treated with drugs as indicated. At the end of treatment, cells were harvested by pooling detached cells and gently disaggregating adherent cells by trypsinisation from each well, pelleted and washed once by centrifugation at 700g for 5 min, resuspended in 250 µl of PBS. 5µl of 100 µg ml-1 Propidium Iodide (SIGMA, Cat# P4170) solution in PBS was added to each sample, mixed well, incubated for 10min, and measured on a BD Fortessa™ Flow Cytometer. Cells positive for PI were considered terminally dead, and PI negative cells as live. BD FACS Diva and FlowJo V7.6.5 and V10.1.1 software packages were used for data analysis.<br><br>For EdU-incorporation cell cycle analysis, cells were pulse-labelled with 10 µM 5′-ethynyl-2′-deoxyuridine (EdU) (Jena Biosciences, Cat# CLK-N001) for 15 min, harvested, washed in 1% BSA-PBS, sequentially fixed in 4% paraformaldehyde and ice-cold 70% ethanol and stored at -20°C. To stain mitotic cells, samples were washed in 1% BSA-PBS, permeabilized with 0.25% Triton-X100 in PBS for 20 min, blocked in 5% FCS in PBS, 30 min, incubated with pSer10 Histone H3-AlexaFlour® 647 conjugate (BioLegend, Cat# 650806), 1-2 h and washed in 1% BSA-PBS. EdU incorporation was detected by copper-coupled Click-iT chemistry (Cu-CCC) as previously described (51, 52). Cells were incubated with freshly prepared Click-iT reaction cocktail (50 mM Tris-HCl pH7.6, 150 mM NaCl, 4 mM CuSO4, 1 µM Picolyl Azide 5/6-FAM (Jena Bioscience, Cat# CLK-1180)), 2 mg ml-1 Sodium-L-Ascorbate (SIGMA, #A4034) for 30 min at RT. Cells were washed and resuspended in 1% BSA-PBS with 0.5 µg ml-1 DAPI for 30 min and measured on a BD LSR FORTESSA Flow Cytometer (BD Biosciences). BD FACS Diva and FlowJo V7.6.5 and V10.1.1 software packages were used for data analysis.
Instrument	BD LSR Fortessa-II
Software	FlowJo V7.6.5 and V10.1.1
Cell population abundance	Not applicable. No cell sorting performed in this study.
Gating strategy	For PI exclusion cell death assays, representative histogram plots in Fig. 3e clarify gating strategy.<br><br>For EdU-incorporation cell cycle analysis in Supplementary Fig. 5d, single cells were gated from the cell population by plotting DNA content area versus width i.e, DAPI-A (area; X-axis) against DAPI-W (width; Y-axis). Dotplots presented in Suppl. Fig. 5d show at least 20000 single cells.

☒ Tick this box to confirm that a figure exemplifying the gating strategy is provided in the Supplementary Information.
